# Small intestine dysfunction in Parkinson’s disease

**DOI:** 10.1007/s00702-015-1442-0

**Published:** 2015-08-26

**Authors:** Justyna Dutkiewicz, Stanisław Szlufik, Michał Nieciecki, Ingeborga Charzyńska, Leszek Królicki, Piotr Smektała, Andrzej Friedman

**Affiliations:** Department of Neurology, Faculty of Heath Science, Medical University of Warsaw, ul. Kondratowicza 8, 03-242 Warsaw, Poland; Department of Diagnostic Imaging, Mazovian Brodno Hospital in Warsaw, ul. Kondratowicza 8, 03-242 Warsaw, Poland; Faculty of Materials Science and Engineering, Warsaw University of Technology, ul. Woloska 141, 02-507 Warsaw, Poland

**Keywords:** Parkinson’s disease, Small intestine passage, Autonomic dysfunction

## Abstract

The aim of this study was to assess the small bowel transit time in patients with Parkinson’s disease (PD). Ten patients with PD with no gastrointestinal complaints and ten healthy control subjects were investigated using single photon emission computed tomography fused with computed tomography after swallowing of a specially prepared capsule containing technetium 99m, which allowed visualization of the passage in the intestines. Preliminary results show that the small intestine passage in PD patients was prolonged compared to controls.

## Introduction

Parkinson’s disease (PD) is one of the most common neurodegenerative disorders. It is characterized by resting tremor, rigidity, bradykinesia and postural instability. Motor symptoms are usually accompanied by non-motor ones including symptoms from the gastrointestinal tract. Constipation was already described by James Parkinson (1817). Gastrointestinal dysfunction is the most common non-motor symptom observed in PD with constipation being the most prevalent, often preceding the motor symptoms (Edward et al. 1992; Berrios et al. 1995; Goldman and Postuma 2014; Krygowska-Wajs et al. 2000). Constipation is five times more common in patients with PD than in general population (Albanese et al. 1997). Defecatory anorectal dysfunctions were found in more than 60 % patients with PD (Edward et al. 1991; Cersosimo and Benarroch 2012). Gastrointestinal symptoms in PD were studied extensively but were aimed mostly on dysphagia, gastric emptying and constipation. Decreased dysphagia limit, prolongation of lower esophageal phase of swallowing and prolongation of laryngeal movement were found in PD patients (Potulska et al. 2003). Gastric emptying delay was detected long time ago (Krygowska-Wajs et al. 2009). Despite all these observations to the best of our knowledge, the function of small intestine was not studied in PD. In the literature, several methods for assessment of the small intestine transit time can be found, such as breath stable-isotope small bowel transit time, radiopaque marker small bowel transit time, breath hydrogen test, inulin or lactulose breath test, small intestine scintigraphy. Small bowel transit time can be also measured with wireless motility capsule (Lawrence et al. 2012). Unfortunately, indigestible solid particles such as the radiopaque markers and wireless motility capsule may not move through the gastrointestinal tract in the same manner as a physiologic meal (Madsen et al. 1991). For breath test methods, the oro-cecal transit time can be misassessed in patients with gastroparesis (Lawrence et al. 2012). The aim of this study was to investigate dynamic of small intestine transport using single photon emission computed tomography fused with computed tomography (SPECT/CT) examination in patients with Parkinson disease, who had to swallow a small size capsule filled with radioisotope. The use of human subjects was carried out with adequate understanding and written consent of the subjects. This study was approved by the Ethics Committee of the Medical University of Warsaw.

## Materials and methods

**Fig. 1:**
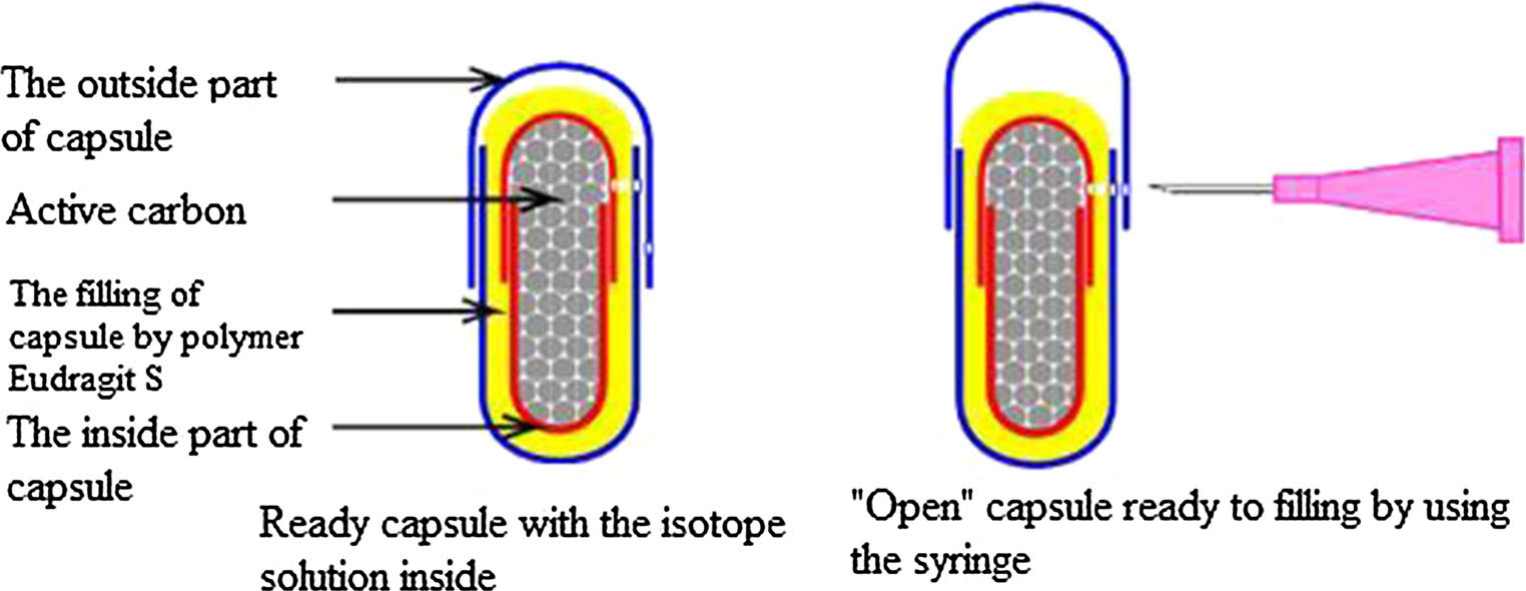
The capsule used in this experiment

**Fig. 2:**
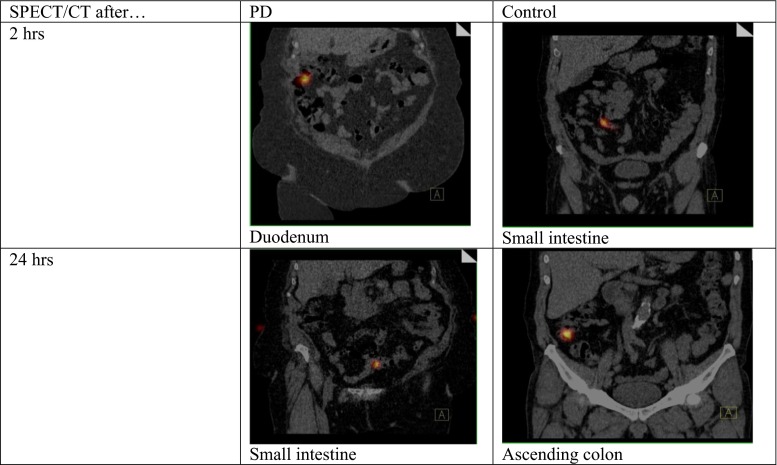
Typical picture of the fusion of SPECT and CT in PD and control subjects after 2 and 24 h after the intake of the capsule containing the isotope

Ten patients with Parkinson’s disease lasting for 6–12 years fulfilling international criteria of the diagnosis, at the ages ranging from 45 to 70, of both sexes (6 females and 4 males), with no gastrointestinal complaints, treated with levodopa only (average daily dose 965 ± 295 mg) were studied and compared to ten control age and sex-matched subjects (more details in Table 1). Patients from control group were hospitalized in our clinic for other reasons than Parkinson’s disease. Subjects in both groups were not receiving drugs that might affect the motility of the gastrointestinal tract. The PD patients were not receiving any antiparkinsonian medication starting 12 h before the swallow until the end of the study. The capsule containing the isotope ^99m^Tc-colloid, which was able to pass through the whole gut without disintegration (Fig. 1) was swallowed in the morning on empty stomach and during the day on which the tests were performed all subjects had regular meals prepared in the hospital. Single photon emission computed tomography (SPECT) and computed tomography (CT) images obtained 2, 4, 6, 8 and 24 h after intake of the capsule were fused. The fusion of SPECT and CT is shown in Fig. 2.

**Table 1:** Data of patients

	PD group	Control group
Number	10	10
Sex F/M	6/4	4/6
Age (years ± SD)	60 ± 8.4	60 ± 8.6
Levodopa mg (daily dose ± SD)	965 ± 295	Without
Drugs that may affect the motility of the gastrointestinal tract	Without	Without

## Results

The appearance of the capsule in small intestine was delayed in PD patients compared to control as it was present in this segment of the gut already after 2 h after the intake in five controls and only in 2 PD patients. In all control subjects, the capsule left small intestine within 4 h. In seven PD patients, the capsule was present in the small intestine for longer than 4 h being seen in one case also after 24 h. This last difference is statistically significant (*p* < 0.01) as determined by *χ*^2^ test.

## Discussion

Most of the methods for the examination of small bowel transit time are expensive, time-consuming, and what is most important have not gained widespread use because of the lack of standardized methods for the studies. It is not known if prolongation of oro-cecal transit time is due only to dysfunction in upper part of gastrointestinal system and large bowel or if a dysfunction of small bowel plays a role in this. Some researches deduced that prolongation of the oropharyngeal transit time is caused by rigidity and hypokinesia, and these symptoms affect the motor function of the tongue (Bushmann et al. 1989). The mechanism of swallowing disorders in PD may be the result of degeneration of the dorsal nucleus of the vagal nerve and esophageal myenteric plexus (Wakabayashi and Takahashi 1997). Pouclet et al. (2012) suggest that the Lewy pathology in gastrointestinal tract in PD patients is the cause of the elongated transit time. Our study has shown that elongation of the oro-cecal transit time in patients with Parkinson’s disease may be related not only to the dysfunction of the upper gastrointestinal tract and colon, but also to the small intestine dysfunction. The data obtained from the study give physicians a different perspective on the problem of constipation in PD.

In conclusion, our preliminary results show that in PD there is a slowing down of the small intestine passage even in patients without any gastrointestinal clinical symptoms. This finding could influence the treatment of constipation in PD.
